# Meningitis in critically ill patients admitted to intensive care unit for severe community-acquired pneumococcal pneumonia

**DOI:** 10.1186/s13613-023-01211-z

**Published:** 2023-12-18

**Authors:** Paul Jaubert, Julien Charpentier, Sarah Benghanem, Alain Cariou, Frédéric Pène, Jean-Paul Mira, Mathieu Jozwiak

**Affiliations:** 1 Service de Médecine Intensive Réanimation, Hôpitaux Universitaires Paris Centre, Hôpital Cochin, Assistance Publique–Hôpitaux de Paris, 27 Rue du Faubourg Saint Jacques, 75014 Paris, France; 2 Université de Paris Cité, Paris, France; 3 UR2CA - Unité de Recherche Clinique Côte d’Azur, Université Côte d’Azur, Nice, France

**Keywords:** Prognosis, Lumbar puncture, Neurological sequelae, Mortality, Streptococcus pneumoniae

## Abstract

**Background:**

Although it has been reported that patients with pneumococcal pneumonia may develop meningitis, lumbar puncture is not systematically recommended in these patients, even in patients with associated bacteremia or invasive pneumococcal disease. The aim of this study was to determine the characteristics and outcomes of patients admitted to intensive care unit (ICU) for pneumococcal community-acquired pneumonia who developed meningitis.

**Methods:**

We retrospectively included all consecutive patients admitted to our ICU from January 2006 to December 2020 for severe pneumococcal community-acquired pneumonia according to American Thoracic Society criteria. Meningitis was defined as pleocytosis > 5 cells/mm^3^ or a positive culture of cerebrospinal fluid for *Streptococcus pneumoniae* in lumbar puncture. The primary endpoint was the proportion of patients with meningitis during their ICU stay.

**Results:**

Overall, 262 patients [64(52–75) years old] were included: 154(59%) were male, 80(30%) had chronic respiratory disease, 105(39%) were immunocompromised and 6(2%) were vaccinated against *S. pneumoniae*. A lumbar puncture was performed in 88(34%) patients with a delay from ICU admission to puncture lumbar of 10.5 (2.8–24.1) h and after the initiation of pneumococcal antibiotherapy in 81(92%) patients. Meningitis was diagnosed in 14 patients: 16% of patients with lumbar puncture and 5% of patients in the whole population. Patients with meningitis had more frequently human immunodeficiency virus positive status (29 vs. 5%, *p* = 0.02), neurological deficits on ICU admission (43 vs. 16%, *p* = 0.03) and pneumococcal bacteremia (71 vs. 30%, *p* < 0.01) than those without. The ICU mortality rate (14 vs. 13%, *p* = 0.73) and the mortality rate at Day-90 (21 vs. 15%, *p* = 0.83) did not differ between patients with and without meningitis. The proportion of patients with neurological disorders at ICU discharge was higher in patients with meningitis (64 vs. 23%, *p* < 0.001) than in those without. The other outcomes did not differ at ICU discharge, Day-30 and Day-90 between the two groups of patients.

**Conclusion:**

Meningitis was diagnosed in 16% of patients with severe pneumococcal community-acquired pneumonia in whom a lumbar puncture was performed, was more frequent in patients with pneumococcal bacteremia and was associated with more frequent neurological disorders at ICU discharge. Further studies are needed to confirm these results.

**Supplementary Information:**

The online version contains supplementary material available at 10.1186/s13613-023-01211-z.

## Background

Community-acquired pneumonia is the first cause of admission to intensive care unit (ICU) and mortality from infectious disease [1–5]. *Streptococcus pneumoniae* is the principal causative agent of community-acquired pneumonia requiring ICU admission [3, 5] and can be responsible for acute respiratory distress syndrome and invasive pneumococcal disease [6] with a high mortality rate [3, 7–9].

It has been reported that patients with pneumococcal pneumonia may develop meningitis and it has been hypothesized that meningitis could be related to hematogenous dissemination and thus probably more frequent in patients with invasive pneumococcal disease [6, 10]. However, up to 30–50% of patients admitted to ICU for pneumococcal pneumonia may have non-specific neurological signs related to septic-associated encephalopathy [11, 12], such as disorders of consciousness ranging from delirium to coma [13]. Currently, lumbar puncture is not systematically recommended in patients with pneumococcal pneumonia, even in patients with associated bacteremia or invasive pneumococcal disease [14]. It could be therefore difficult to select patients in whom meningitis should be suspected and perform a lumbar puncture to confirm the diagnosis, while the management of patients with pneumococcal pneumonia would differ in case of associated meningitis, notably by administering antibiotics at meningeal doses. In addition, the mortality rate and prognosis of these patients may be affected if appropriate management is delayed [15].

To date, the issue of meningitis in patients admitted to ICU for pneumococcal community-acquired pneumonia has been scarcely studied. The aim of this study was to determine the characteristics and outcomes of patients with pneumococcal community-acquired pneumonia who developed meningitis.

## Methods

### Study design

This observational and retrospective study was conducted in a 24-bed ICU of a French university hospital. The study was approved by the Ethics committee of the French Intensive Care Society (CE-SRLF 23-046). Informed consent was waived, but all patients or next-of-kin were informed about the study. The study complied with the Strengthening the Reporting of Observational Studies in Epidemiology (STROBE) statement guidelines [16].

### Patients

We included all consecutive patients over 18 years old admitted to our 24-bed ICU for severe pneumococcal community-acquired pneumonia according to *American Thoracic Society* criteria from January 2006 to December 2020. Exclusion criteria were patients admitted to ICU for pneumococcal infection other than pneumonia and patients under legal protection. The onset of the study period corresponded to the implementation in our unit of our computerized data collection software (Clinisoft^™^, GE Healthcare Centricity, Chicago, IL, United States).

### Definition of severe pneumococcal community-acquired pneumonia

Community-acquired pneumonia was defined by the presence on hospital admission or within 48 h of admission to hospital of one or more of the following features, according to the adapted *American Thoracic Society* criteria [17]: new or increased cough with or without sputum production, tachypnoea, chest pain, abnormal temperature (> 38 °C or < 36 °C), lung consolidation on physical examination, and a new radiological infiltrate.

The pneumonia severity was also defined according to the *American Thoracic Society* criteria [17], requiring either one of two major criteria (need for invasive mechanical ventilation or septic shock) or any three minor criteria: respiratory rate > 30/min, confusion or disorientation, body temperature < 36 °C, arterial hypotension requiring fluid administration, multilobar radiological infiltrates, partial arterial oxygen pressure over inspired oxygen fraction (PaO_2_/FiO_2_) ratio ≤ 250, blood urea nitrogen ≥ 20 mg/dL, leukocytes < 4000/mm^3^, or platelets < 100,000/mm^3^.

Microbiological samples considered for diagnosis were the following: positive urinary antigen, respiratory samples with standard criteria of quality (positive quantitative culture of sputum examination, endotracheal aspirate, broncho-alveolar lavage or protected specimen brush with a bacterial count ≥ 10^7^, 10^6^, 104 or 10^3^ colony forming unit/mL, respectively), positive pleural culture, or a positive blood culture.

### Data collection and endpoints

Demographic characteristics, comorbidities of patients, clinical, biological, electroencephalographic, and brain imaging data were collected and analyzed, as well as therapeutics and clinical outcomes. Outcomes were assessed at ICU discharge, Day-30 and Day-90 or until patients’ death if it occurred before Day-90. At ICU discharge, the patients' degree of neurological disability was assessed using the Glasgow Outcome Scale (GOS), which ranges from 1 (death) to 5 (good recovery) [18]. Good neurological outcome was defined as GOS 4–5 and poor outcome as GOS 1–3. At Day-30 and Day-90, the patients’ status was assessed from the medical records.

The primary endpoint was the proportion of patients with meningitis during their ICU stay. The secondary endpoints were characteristics of patients with lumbar puncture, the risk factors for meningitis, the mortality rate, the duration of mechanical ventilation, the ICU length of stay and the proportion of patients with neurological disorders.

Meningitis was defined as pleocytosis > 5 cells/mm^3^ [19, 20] and/or a positive culture of cerebrospinal fluid for *S. pneumoniae* in lumbar puncture. In case of traumatic lumbar puncture, one white blood cell was subtracted for every 500 red blood cells to avoid an artificial increase in white blood cells’ count [21].

Neurological deficits were defined by the presence on ICU admission or the occurrence during ICU stay of one or more of the following signs: sensitivity-motor deficits, pyramidal syndrome, cranial nerve palsy, clinical seizure, or meningeal syndrome (headache, neck stiffness, altered mental status, and Kernig or Brudzinski signs).

Neurological disorders were defined as the persistence of at least one of the above defined neurological deficits at ICU discharge.

### Statistical analysis

Continuous variables were expressed as median (interquartile range) and categorical variables as numbers (percentages). Between-group comparisons were performed by Mann–Whitney tests for continuous variables and by Pearson’s Chi-square or Fisher’s exact tests for categorical variables. Analyses were performed with R 3.1.1 (R foundation for Statistical Computing Vienna, Austria). Descriptive statistics were only carried out on the available data. The percentage of missing data for each variable is detailed in Additional file 1: Table S1. All tests were two-sided, and a *p* < 0.05 was considered statistically significant.

## Results

### Patients

**Fig. 1:**
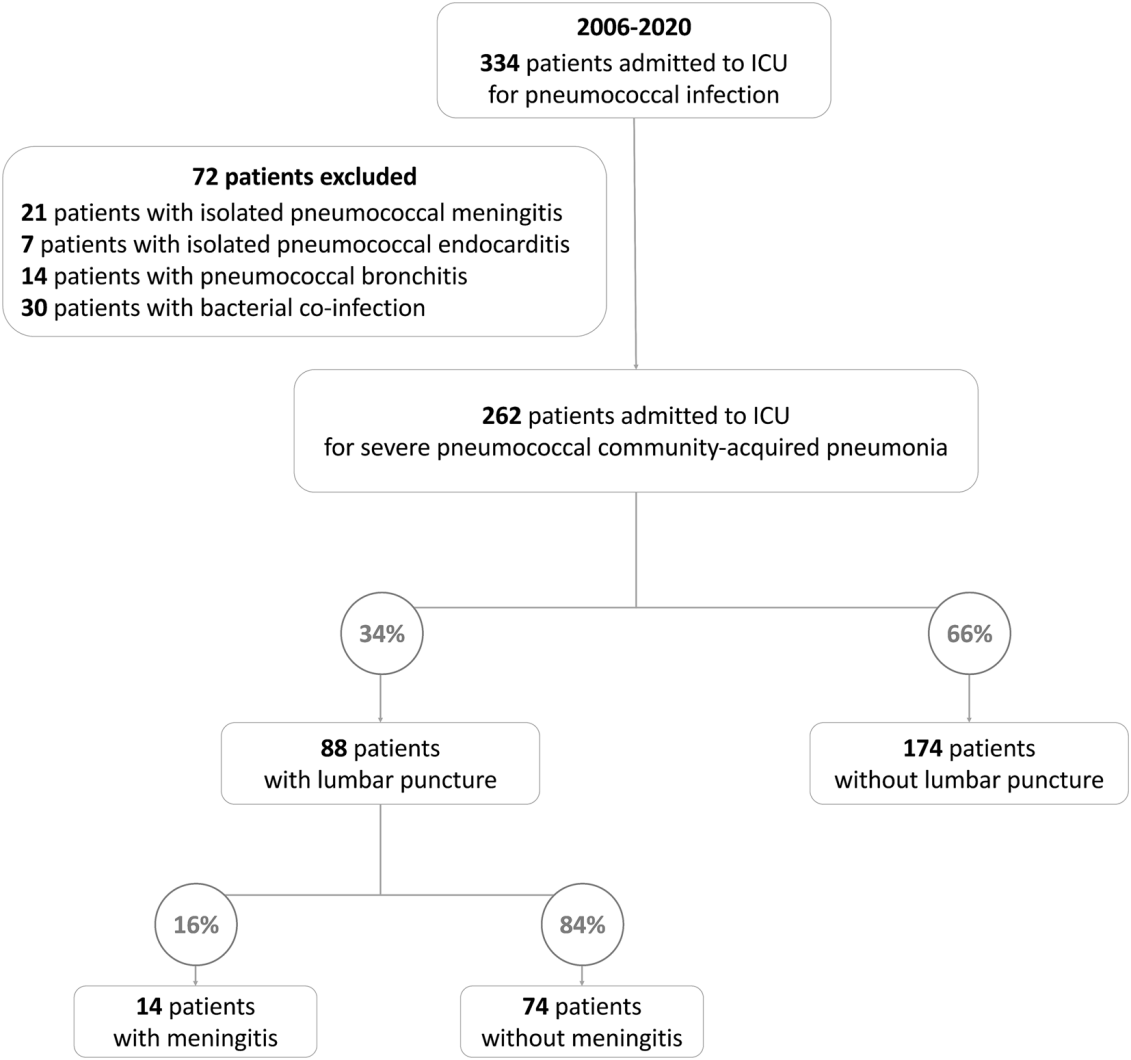
Flowchart of the study. *ICU* intensive care unit

Between 2006 and 2020, 336 patients were admitted to ICU for a pneumococcal infection and 262 were included in the final analysis (Fig. 1). Overall, patients were 64(52–75) years old, 154(59%) were male, 80(30%) had chronic respiratory disease, 105(39%) were immunocompromised and 6(2%) were vaccinated against *S. pneumoniae*. During ICU stay, 78(30%) patients developed septic shock, and 155(59%) patients required invasive mechanical ventilation with a ventilation duration of 8(5–15) days. The ICU mortality rate was 16%.

### Lumbar puncture during ICU stay

No lumbar puncture was performed prior to ICU admission, and a lumbar puncture was performed in 88(34%) patients during their ICU stay with a delay from ICU admission to lumbar puncture of 10.5(2.8–24.1) h. In 81(92%) patients, the lumbar puncture was performed after the initiation of pneumococcal antibiotherapy: Cefotaxime in 48(55%) patients, Amoxicillin in 4(4%) patients, Amoxicillin-Clavulanate in 20(23%) patients and Piperacillin-Tazobactam in 9(10%) patients.

**Fig. 2:**
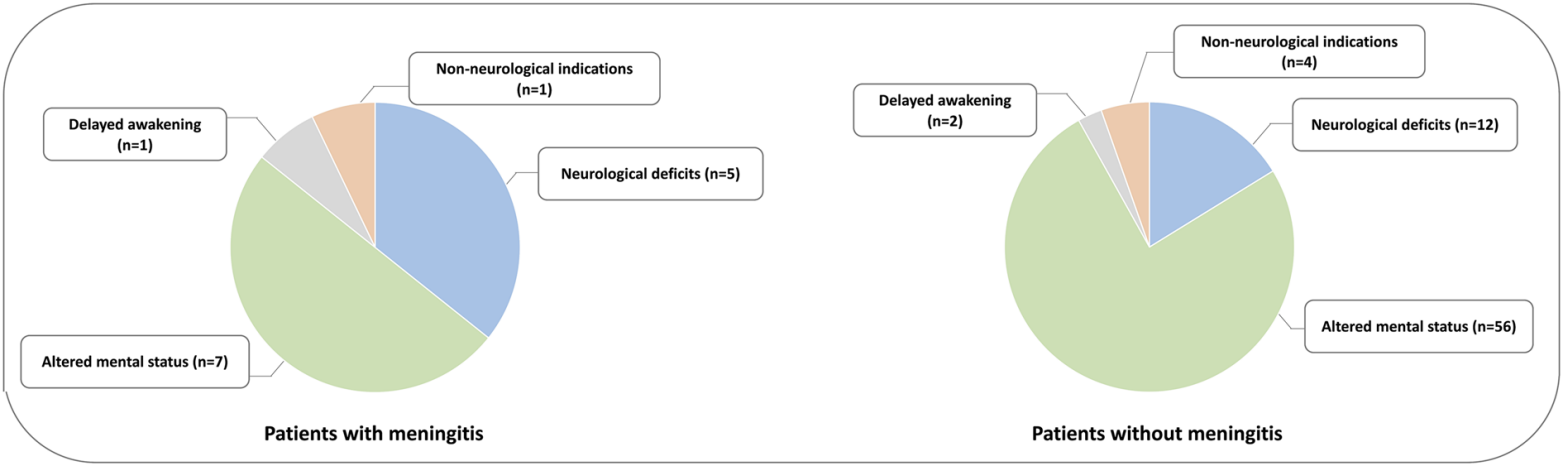
Indications of lumbar puncture during intensive care unit stay in patient with (*n* = 14) and without (*n* = 74) meningitis

There was no predefined indication to lumbar puncture, which was left to the discretion of the attending physician. Lumbar puncture was performed for altered mental status in 63(72%) patients, neurological deficits in 17(19%) patients, delayed awakening in 3(3%) patients and non-neurological indications in 5(6%) patients (Fig. 2). No lumbar puncture was performed for pneumococcal bacteremia. Patients with a lumbar puncture were more severe, had more frequently a medical history of alcohol abuse, had a lower Glasgow coma scale on ICU admission (11(3–15) *vs.* 15(14–15), *p* < 0.0001), had more frequently neurological deficits on ICU admission (20 *vs.* 3%, *p* < 0.0001), had less frequently a positive urinary antigen only (18 *vs.* 40%, *p* < 0.001) and more frequently positive respiratory samples (70 *vs.* 45%, *p* < 0.001) than those without (Table 1). The proportion of patients with pneumococcal bacteremia was not different between patients with and without lumbar puncture (36 *vs.* 28%, *p* = 0.22) (Table 1).

**Table 1:** Patient characteristics and ICU management in the whole population

Variables	No lumbar puncture (*n* = 174)	Lumbar puncture (*n* = 88)	*p* value
Demographic characteristics			
Age (years)	65 (53–77)	59 (49–74)	0.25
Male gender, *n* (%)	97 (56)	57 (65)	0.20
Underlying conditions			
Smokers, *n* (%)	89 (51)	43 (49)	0.83
Alcohol abuse, *n* (%)	35 (20)	31 (35)	0.01
Obesity, *n* (%)	16 (9)	7 (8)	0.74
Diabetes mellitus, *n* (%)	21 (12)	11 (12)	0.92
Asplenia, n (%)	3 (2)	1 (1)	0.87
Human immunodeficiency virus, *n* (%)	8 (5)	8 (9)	0.24
Immunosuppressive treatments, *n* (%)	31 (18)	7 (8)	0.03
Pneumococcal vaccination, *n* (%)	4 (2)	2 (2)	0.67
Clinical characteristics in ICU			
SAPS-2 score	47 (35–69)	62 (42–81)	< 0.01
SOFA score on ICU admission	5 (3–9)	7 (5–12)	< 0.001
Charlson score	1 (0–3)	1 (0–3)	0.72
Body mass index (kg/m^2^)	22 (20–26)	24 (20–27)	0.83
Glasgow coma scale on ICU admission	15 (14–15)	11 (3–15)	< 0.0001
Neurological deficits on ICU admission, *n* (%)	5 (3)	18 (20)	< 0.0001
Pneumococcal antibiotherapy prior to ICU admission, *n* (%)	86 (49)	38 (43)	0.41
Septic shock, *n* (%)	38 (22)	40 (45)	< 0.001
ARDS, *n* (%)	82 (47)	73 (83)	< 0.001
Biological variables on ICU admission			
Leukocytes (× 10^9^/L)	12.9 (7.7–18.6)	11.9 (5.1–17.1)	0.17
Lymphocytes (× 10^9^/L)	0.6 (0.3–1.0)	0.7 (0.4–1.1)	0.43
CD4 (× 10^9^/L)*	267 (43–430)	31 (14–60)	0.31
Platelets (× 10^9^/L)	201 (137–265)	185 (107–257)	0.23
Procalcitonin (ng/L)	10.2 (1.9–47.6)	10.6 (0.8–25.9)	0.49
Arterial blood lactate level (mmol/L)	2.1 (1.3–3.3)	2.2 (1.5–3.5)	0.57
Microbiological diagnosis			
Positive urinary antigen, *n* (%)	141 (81)	73 (83)	0.83
Positive urinary antigen only, *n* (%)	69 (40)	16 (18)	< 0.001
Positive respiratory samples (%)	79 (45)	62 (70)	< 0.001
Positive blood culture, *n* (%)	49 (28)	32 (36)	0.22
Management during ICU stay			
Intubation, *n* (%)	82 (47)	73 (83)	< 0.0001
Renal replacement therapy, *n* (%)	28 (16)	15 (17)	0.84
Neurological disorders, *n* (%)	12 (7)	26 (30)	< 0.0001
Ventilator-associated pneumonia, *n* (%)	20 (11)	20 (23)	0.03
Duration of invasive mechanical ventilation (days)	8 (5–13)	9 (6–20)	0.10
ICU length of stay (days)	4 (2–8)	10 (6–23)	< 0.0001

Data are expressed as median (interquartile range) or counts (percentages)

*ARDS* acute respiratory distress syndrome, *ICU* intensive care unit, *SAPS* simplified acute physiology score, *SOFA* sepsis-related organ failure assessment

*Data available in 10 patients among the 16 patients with human immunodeficiency virus positive status

### Meningitis diagnosis

Meningitis was diagnosed in 14 patients: 16% of patients with lumbar puncture and 5% of patients in the whole population (Fig. 1). Patients with meningitis had more frequently human immunodeficiency virus positive status (29 vs. 5%, *p* = 0.02), neurological deficits on ICU admission (43 vs. 16%, *p* = 0.03) and pneumococcal bacteremia (71 vs. 30%, *p* < 0.01) than those without. The Glasgow coma scale (9(3–14) vs. 12(3–15), *p* = 0.31) did not differ between the two groups of patients (Table 2). Cerebrospinal fluid examination showed a higher protein level [0.9(0.6–3.7) vs. 0.3(0.3–0.4) g/L, *p* < 0.0001] and a lower glucose concentration [3.3(0.1–4.6) vs. 4.7(3.8–5.0) mmol/L, *p* = 0.01] in patients with meningitis than in patients without.

**Table 2:** Patient characteristics and ICU management according to the diagnosis of meningitis

Variables	No meningitis (*n* = 74)	Meningitis (*n* = 14)	*p* value
Demographic characteristics			
Age (years)	60 (50–74)	55 (48–70)	0.57
Male gender, *n* (%)	50 (68)	7 (50)	0.34
Underlying conditions			
Smokers, n (%)	39 (53)	4 (29)	0.17
Alcohol abuse, *n* (%)	27 (36)	4 (29)	0.79
Obesity, *n* (%)	6 (8)	1 (7)	1.00
Diabetes mellitus, *n* (%)	11 (15)	0 (0)	0.27
Asplenia, *n* (%)	1 (1)	0 (0)	0.35
Human immunodeficiency virus, *n* (%)	4 (5)	4 (29)	0.02
Immunosuppressive treatments, *n* (%)	7 (9)	0 (0)	0.58
Pneumococcal vaccination, *n* (%)	2 (3)	0 (0)	0.72
Clinical characteristics in ICU			
SAPS-2 score	64 (42–81)	50 (30–81)	0.54
SOFA score on ICU admission	7 (5–12)	7 (4–14)	0.58
Charlson score	1 (0–3)	0 (0–5)	0.85
Body mass index (kg/m^2^)	23 (20–27)	25 (21–27)	0.69
Glasgow coma scale on ICU admission	12 (3–15)	9 (3–14)	0.31
Neurological deficits on ICU admission, *n* (%)	12 (16)	6 (43)	0.03
Septic shock, *n* (%)	35 (47)	5 (36)	0.61
ARDS, *n* (%)	63 (85)	10 (71)	0.25
Biological variables on ICU admission			
Leukocytes (× 10^9^/L)	12.2 (5.0–7.1)	9.5 (6.6–20.6)	0.69
Lymphocytes (× 10^9^/L)	0.7 (0.4–1.1)	0.5 (0.3–0.9)	0.62
CD4 (× 10^9^/L)*	226 (113–338)	31 (22–45)	N/A
Platelets (× 10^9^/L)	184 (108–256)	197 (105–304)	0.80
Procalcitonin (ng/L)	9.9 (0.7–26.6)	11.2 (1.9–20.2)	0.69
Arterial blood lactate level (mmol/L)	2.0 (1.5–3.3)	2.7 (1.6–4.0)	0.53
Microbiological diagnosis			
Positive urinary antigen, *n* (%)	62 (84)	11 (79)	0.93
Positive urinary antigen only, *n* (%)	15 (20)	1 (7)	0.43
Positive respiratory samples (%)	53 (72)	9 (64)	0.82
Positive blood culture, *n* (%)	22 (30)	10 (71)	< 0.01
Management during ICU stay			
Delay from ICU admission to lumbar puncture (h)	12 (3–24)	5 (1–21)	0.25
Pneumococcal antibiotherapy prior to lumbar puncture, *n* (%)	69 (93)	12 (86)	0.31
Intubation, *n* (%)	63 (85)	10 (71)	0.39
Renal replacement therapy, *n* (%)	12 (16)	3 (21)	0.70
Ventilator-associated pneumonia, *n* (%)	18 (24)	2 (14)	0.63
Duration of invasive mechanical ventilation (days)	9 (5–21)	10 (7–15)	0.83
ICU length of stay (days)	10 (6–24)	8 (3–17)	0.30

Data are expressed as median (interquartile range) or counts (percentages)

*ARDS* acute respiratory distress syndrome, *ICU* intensive care unit, *SAPS* simplified acute physiology score, *SOFA* sepsis-related organ failure assessment

*Data available in five patients among the eight patients with human immunodeficiency virus positive status

In patients with meningitis, the pleocytosis was 33(15–220) cells/mm^3^ with a predominance of neutrophils and 5(36%) patients had a pleocytosis without positive culture of cerebrospinal fluid (all had negative Gram strain). Culture of cerebrospinal fluid allowed *S. pneumoniae* isolation in 9(64%) patients: all but one patient had a pleocytosis, 6 patients had positive Gram strain and 3 patients had negative Gram strain. The proportion of patients receiving pneumococcal antibiotherapy prior to lCU admission (57 vs. 40%, *p* = 0.39) and prior to lumbar puncture (86 vs. 93%, *p* = 0.31) did not differ between patients with and without meningitis (Table 2). All patients with meningitis received meningeal doses of antibiotics and 5(36%) patients received dexamethasone within 1.0 (0.0–1.5) h of lumbar puncture.

### Neurological complementary exams and outcomes

**Fig. 3:**
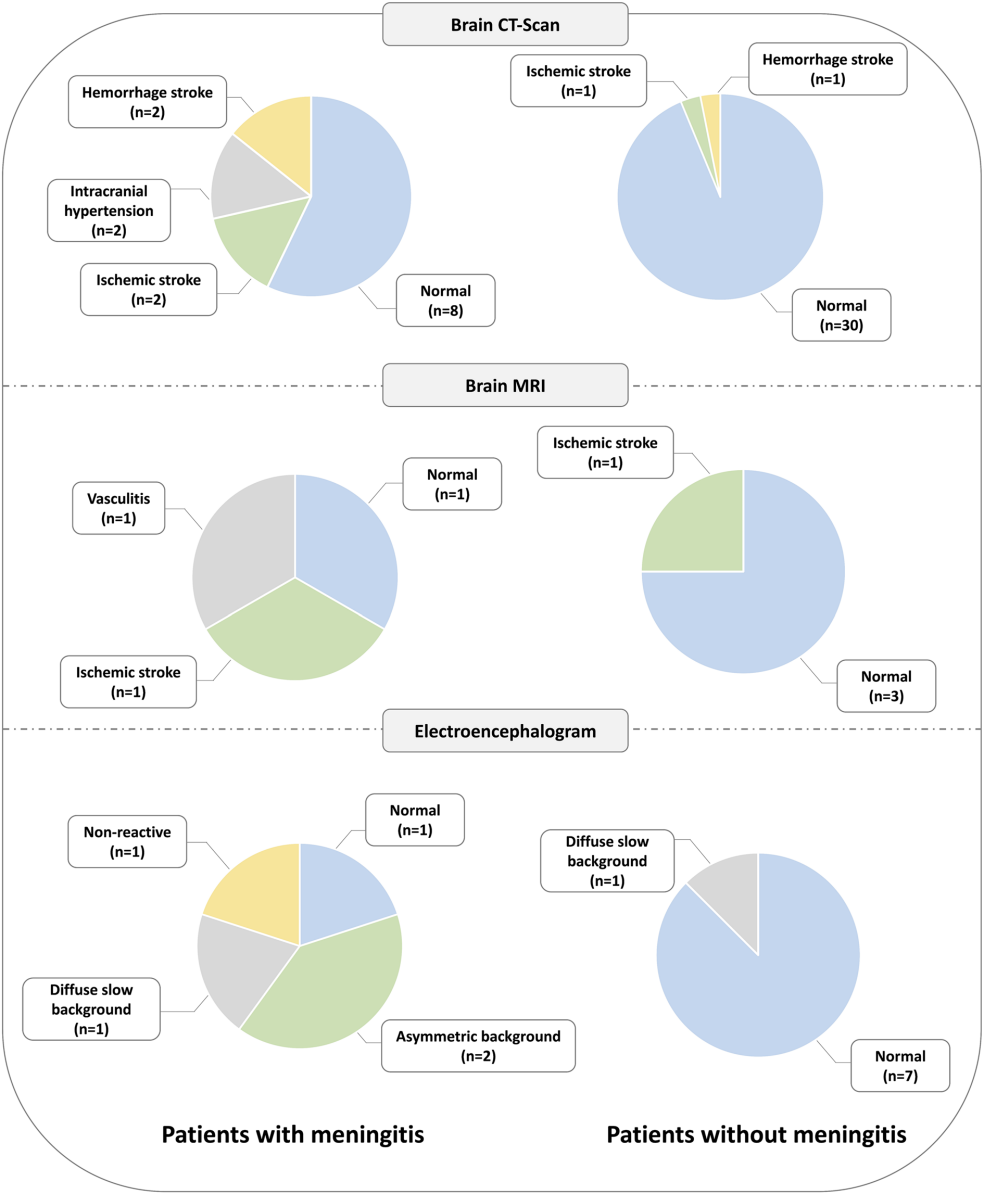
Patterns observed for the different neurological complementary exams in patients with (*n* = 14) and without (*n* = 74) meningitis. *CT-Scan* computed tomography, *MRI* magnetic resonance imaging, *EEG* electroencephalogram

Neurological complementary exams were performed in 44 (50%) patients with lumbar puncture: before lumbar puncture in 19(22%) patients and after lumbar puncture in 25(28%) patients. A non-injected brain CT-scan was systematically performed before lumbar puncture in patients with neurological deficits. Overall, 16% of brain CT-Scan and 100% of magnetic resonance imaging were performed with contrast injection. Among the seven patients who had brain magnetic resonance imaging, 5 (71%) had also brain CT-Scan. During ICU stay, 40 (54%) patients without meningitis had no neurological complementary exams and all patients with meningitis had at least one neurological complementary exam. Brain CT-Scan (100 *vs.* 43% *p* < 0.01) and electroencephalogram (36 *vs.* 11%, *p* = 0.04), but not brain magnetic resonance imaging (21 *vs.* 5%, *p* = 0.13), were more frequently performed in patients with meningitis than in those without. In patients with meningitis, brain CT-Scan was considered as abnormal in 6 (43%) patients, with intracranial hypertension in 2 (14%) patients, ischemic stroke in 2 (14%) patients and hemorrhage stroke in 2 (14%) patients. Brain magnetic resonance imaging found cerebral vasculitis in one patient, ischemic stroke in one patient and was considered normal in one patient. Electroencephalogram was abnormal in 4 (80%) patients, with asymmetric background in 2 (40%) patients, diffuse slow background in 1 (20%) patient and non-reactive electroencephalogram in one patient (Fig. 3).

The mortality rates at ICU and Day-30 (14 *vs.* 13%, *p* = 0.73), at hospital discharge (27 *vs.* 19%, *p* = 0.69) and at Day-90 (21 *vs.* 15%, *p* = 0.83) did not differ between patients with and without meningitis. Causes of mortality in the three deceased patients with meningitis were brain death, post-cardiac arrest syndrome and withdrawal of life-sustaining therapy for non-neurological reason. At ICU discharge, the proportion of patients with a GOS ≥ 4 did not differ between the two groups of patients, but the proportion of patients with neurological disorders was higher in patients with meningitis (64 *vs.* 23%, *p* < 0.001) than in those without (Table 3). Among patients with meningitis, 7 (50%) patients had persistent altered mental status, 1 (7%) patient had sensitivity-motor deficit, and 1 (7%) patient had epilepsy at ICU discharge. Among patients without meningitis, 13 (18%) patients had persistent altered mental status, 4 (5%) patients had sensitivity-motor deficit, and none had epilepsy at ICU discharge. The other outcomes did not differ at ICU discharge, hospital discharge, Day-30 and Day-90 between the two groups of patients (Table 3).

**Table 3:** Outcomes of patients according to the diagnosis of meningitis

Variables	No meningitis (*n* = 74)	Meningitis (*n* = 14)	*p* value
At ICU discharge			
Mortality rate, *n* (%)	10 (13)	2 (14)	0.73
Neurological disorders, *n* (%)	17 (23)	9 (64)	< 0.001
Glasgow Outcome Scale (1–3), *n* (%)	24 (32)	4 (29)	1.00
Glasgow Outcome Scale (4–5), *n* (%)	50 (68)	10 (71)	1.00
At hospital discharge*			
At home, *n* (%)	38 (57)	5 (45)	0.53
Rehabilitation, *n* (%)	16 (24)	3 (27)	1.00
Mortality rate, *n* (%)	13 (19)	3 (27)	0.69
At Day-30*			
At home, *n* (%)	26 (37)	3 (23)	0.53
Rehabilitation, *n* (%)	3 (4)	0 (0)	0.97
Hospitalized outside ICU, *n* (%)	20 (28)	5 (39)	0.52
Hospitalized in ICU, *n* (%)	12 (17)	3 (23)	0.69
Mortality rate, *n* (%)	10 (14)	2 (15)	1.00
At Day-90*			
At home, *n* (%)	43 (71)	4 (50)	0.42
Rehabilitation, *n* (%)	2 (3)	0 (0)	1.00
Hospitalized outside ICU, *n* (%)	3 (5)	1 (13)	0.39
Hospitalized in ICU, *n* (%)	2 (3)	0 (0)	1.00
Mortality rate, *n* (%)	11 (18)	3 (37)	0.19

Data are expressed as counts (percentages)

The Glasgow Outcome Scale ranges from 1 (death) to 5 (good recovery)

*ICU* intensive care unit

*missing data : 10 missing data at hospital discharge, 4 missing data at Day-30 and 19 missing data at Day-90.

## Discussion

In our cohort of patients admitted to ICU for severe pneumococcal community-acquired pneumonia, 34% of patients had a lumbar puncture during ICU stay and no lumbar puncture was performed for pneumococcal bacteremia. A meningitis was diagnosed in 16% of them and the culture of cerebrospinal fluid allowed *S. pneumoniae* isolation in 64% of patients. Patients with meningitis had more frequently human immunodeficiency virus positive status, neurological deficits on ICU admission, and pneumococcal bacteremia during their ICU stay. Patients with meningitis were more likely to have neurological disorders at ICU discharge than patients without meningitis, despite a similar mortality rate and no different proportion of patients with a GOS ≥ 4.

Although previous studies did not report the proportion of lumbar puncture and therefore meningitis in patients admitted to ICU for pneumococcal pneumonia [7, 9, 11], a mortality rate from neurological cause from 5 to 7% was reported [9, 11, 22], suggesting that some of these patients may have undiagnosed meningitis. In this regard, Figueiredo and colleagues found that in patients admitted for bacterial meningitis, mainly pneumococcal meningitis, pneumonia was a frequent coexisting infection on admission [23].

In our cohort, a lumbar puncture was performed in 34% of patients during ICU stay. The two main indications were altered mental status (from delirium to coma) and neurological deficits on ICU admission. Overall, a meningitis was diagnosed in 16% of patients with lumbar puncture, a proportion similar to the 11% that would been found according to Spano’s nomogram [24]. Culture of cerebrospinal fluid allowed *S. pneumoniae* isolation in 64% of patients, while 92% of patients received pneumococcal antibiotherapy prior to lumbar puncture. The proportion of patients with pneumococcal bacteremia during their ICU stay was not higher in patients with lumbar puncture than in those without, whereas patients with meningitis had more frequently pneumococcal bacteremia during their ICU stay. This suggests that hematogenous dissemination is probably the main mechanism of meningeal invasion in patients admitted to ICU for severe pneumococcal pneumonia, as previously shown for pneumococcal meningitis in experimental and clinical studies [6, 10, 25–27]. We also found that the proportion of patients with a medical history of alcohol abuse was higher in patients with lumbar puncture. This can be explained by the fact that these patients are probably more likely to present altered mental status [28].

The mortality rate of patients admitted to ICU for severe pneumococcal community-acquired pneumonia complicated by meningitis was lower than that of patients admitted to ICU for pneumococcal meningitis [23, 29, 30]. This difference can be explained as follows. First, while most patients with meningitis deceased from neurological causes (mainly refractory intracranial hypertension or stroke) or refractory shock [29–32], all but one patient who developed meningitis in our cohort deceased from non-neurological cause and only 14% of them developed intracranial hypertension on brain CT-Scan during their ICU stay. Second, it cannot be ruled out that the pneumococcal antibiotherapy, even if non-meningeal doses, could be partly efficient. Third, this difference could also be partly explained by the potentially limited meningeal reaction in patients from our cohort, as evidenced by a lower pleocytosis in cerebrospinal fluid than that found in patients hospitalized for pneumococcal meningitis [15, 29, 33]. Nevertheless, this limited meningeal reaction could rather illustrate a limited inoculum size, as evidenced by the low proportion of meningitis patients with negative Gram strain and could also be related to the administration of pneumococcal antibiotherapy prior to lumbar puncture in 92% of patients, even though it was administered in non-meningeal doses. Moreover, the significance of this limited meningeal reaction is subject to debate, with some studies finding that little or no meningeal reaction is a poor prognostic factor in patients with infectious meningitis [19, 29, 34, 35].

Patients with meningitis were more likely to have neurological disorders at ICU discharge, despite a similar ICU mortality rate and no difference in the proportion of patients with a GOS ≥ 4 between patients with and without meningitis. Therefore, even if lumbar puncture was performed in more severe patients (higher SAPS-2 and SOFA scores), patients with meningitis did not appear to be more severe than patients without meningitis. The most frequent neurological disorders were persistent altered mental status. Nevertheless, outcomes at hospital discharge, Day-30 and Day-90 did not appear to differ between patients with and without meningitis, as illustrated by similar mortality rate and proportion of patients at home, in rehabilitation or hospitalized. These results regarding the long-term neurological outcome should be interpreted with caution but may suggest that neurological disorders at ICU discharge could be only transient or due to reversible cause of disabilities, such as post-intensive care syndrome [36]. Our results may also suggest again that pneumococcal meningitis and pneumococcal meningitis secondary to pneumococcal pneumonia are two very different entities regarding pathogenesis and pathophysiology [26], as patients with pneumococcal meningitis are at risk of increased long-term mortality and of developing neurological sequelae leading to disability and dependency [29, 35]. Therefore, Figueiredo and colleagues found that in patients admitted for bacterial meningitis, coexisting pneumonia on admission was independently associated with unfavorable neurological outcomes [23]. As for mortality, the potential efficiency of pneumococcal antibiotherapy in non-meningeal doses may also partly explain these results.

The main clinical implication of our results is that lumbar puncture might be discussed as early as possible in patients admitted to ICU for severe pneumococcal community-acquired pneumonia in case of neurological deficits, altered mental status, pneumococcal bacteremia or with any abnormality (stroke, intracranial hypertension) on neurological complementary exam that could be suggestive of pneumococcal meningitis, as the diagnosis of meningitis requires antibiotic doses to be adjusted, and could therefore also have an impact on patient outcome in cases of delayed diagnosis.

We acknowledge some limitations to our study. First, this was a retrospective study and the incidence of meningitis may have been underestimated, as a lumbar puncture was not systematically performed even in patients with pneumococcal bacteremia or with neurological deficits. Second, we found that patients with human immunodeficiency virus positive status had more frequently meningitis. Nevertheless, the pneumococcal vaccination status of these patients was not available due to the retrospective design of the study, which limits the interpretation of this result. Similarly, we were unable to analyze the impact of steroids in these patients due to missing data on the administration of hemisuccinate in patients with septic shock and the fact that no patients received hemisuccinate for pneumonia during the study period. Third, the diagnosis of intracranial hypertension was made solely by brain CT-Scan. No intracranial pressure monitoring or transcranial Doppler was performed for this purpose. Fourth, we could not perform any multivariate analysis to clearly identify risk factors of meningitis given the relatively small number of patients with meningitis. Fifth, we were not able to assess the proportion of patients with neurological sequelae and the GOS at Day-30 and Day-90 due to the retrospective design of the study. Sixth, the susceptibility of the pneumococcal strains to penicillin G was not available and, therefore, the pneumococcal antibiotherapy could not be confirmed to be entirely appropriate. Nevertheless, the proportion of pneumococcal strains with decreased susceptibility to amoxicillin did not exceed 10% during the study period [7]. Seventh, as lactate measurement and pneumococcal antigen testing were not systematically performed in cerebrospinal fluid during the study period, it was not possible to obtain reliable information on these two parameters in our cohort. Finally, multiplex PCR assay data were not available for cerebrospinal fluid examination during the study period, which may have contributed to underestimating the incidence of meningitis.

## Conclusions

Meningitis was diagnosed in 16% of patients admitted to ICU for severe pneumococcal community-acquired pneumonia in whom a lumbar puncture was performed. Meningitis was more frequent in patients with pneumococcal bacteremia during their ICU stay and was associated with more frequent neurological disorders at ICU discharge. Further studies are needed to confirm these results and to better define the indications of lumbar puncture in these patients.

### Supplementary Information

**Additional file 1: Table S1.** Missing data for each variable in the whole population.

## Data Availability

The datasets used and analyzed during the current study are available from the corresponding author on reasonable request.

## Abbreviations

GOS: Glasgow outcome scale
ICU: Intensive care unit
