# Supplementary material for: Meningitis in critically ill patients admitted to intensive care unit for severe community-acquired pneumococcal pneumonia
Source: Ann Intensive Care. 2023 Dec 18;13:129. doi: 10.1186/s13613-023-01211-z (PMC10728423; doi:10.1186/s13613-023-01211-z)
Supplement: Supplementary file 1 — Additional file 1: Table S1. Missing data for each variable in the whole population. [file 13613_2023_1211_MOESM1_ESM.docx]

**Meningitis in critically ill patients admitted to intensive care unit for severe community-acquired pneumococcal pneumonia**

Paul JAUBERT, MD^1^ ; Julien CHARPENTIER, MD^1^ ; Sarah BENGHANEM, MD^1,2^ ; Alain CARIOU, MD PhD^1,2^ ; Frédéric PENE, MD PhD^1,2^ ; Jean-Paul MIRA, MD PhD^1,2^ ; Mathieu JOZWIAK, MD PhD^1,2,3^

1 : Service de Médecine Intensive Réanimation, Hôpitaux Universitaires Paris Centre, Hôpital Cochin, Assistance Publique – Hôpitaux de Paris, 27 rue du faubourg Saint Jacques, 75014 Paris, France

2 : Université de Paris Cité, Paris, France

3 : UR2CA - Unité de Recherche Clinique Côte d’Azur, Université Côte d’Azur, Nice, France

***Corresponding author***

Mathieu JOZWIAK, MD, PhD

Service de Médecine Intensive Réanimation

Hôpitaux Universitaires Paris Centre, Hôpital Cochin, Assistance Publique – Hôpitaux de Paris 27 rue du faubourg Saint Jacques, 75014 Paris, France

[jozwiak.m@chu-nice.fr](mailto:jozwiak.m@chu-nice.fr)

**Table S1. Missing data for each variable in the whole population.**

|  | Patients  (n=262) |
| --- | --- |
| Demographic characteristics |  |
| Age (years) | 0% (262) |
| Male gender, n (%) | 0% (262) |
|  |  |
| Underlying conditions |  |
| Smokers, n (%) | 0% (262) |
| Alcohol abuse, n (%) | 0% (262) |
| Obesity, n (%) | 9% (238)  )  ) |
| Diabetes mellitus, n (%) | 0% (262) |
| Asplenia, n (%) | 0% (262) |
| Human immunodeficiency virus, n (%) | 0% (262) |
| Immunosuppressive treatments, n (%) | 0% (262) |
| Pneumococcal vaccination | 80% (52) |
|  |  |
| Clinical characteristics in ICU |  |
| SAPS-2 score | 0% (262) |
| SOFA score | 0% (262) |
| Charlson score | 0% (262) |
| Body mass index (kg/m^2^) | 9% (238)  )  ) |
| Glasgow coma scale on ICU admission | 0% (262) |
| Neurological deficits, n (%) | 0% (262) |
| Antibiotherapy prior to ICU admission, n (%) | 0% (262) |
| Septic shock, n (%) | 0% (262) |
| ARDS, n (%) | 0% (262) |
|  |  |
| Biological variables on ICU admission |  |
| Leukocytes (x10^9^/L) | 1% (259) |
| Lymphocytes (x10^9^/L) | 6% (246) |
| CD4 (x10^9^/L)* | 37% (6) |
| Platelets (x10^9^/L) | 1% (259) |
| Procalcitonin (ng/L) | 69% (81) |
| Arterial blood lactate level (mmol/L) | 13% (228) |
|  |  |
| Microbiological diagnosis |  |
| Urinary antigen, n (%) | 0% (262) |
| Respiratory samples (%) | 0% (262) |
| Blood culture, n (%) | 0% (262) |
|  |  |
| Management during ICU stay |  |
| Delay from ICU admission to lumbar puncture (days)** | 9% (80) |
| Intubation, n (%) | 0% (262) |
| Renal replacement therapy, n ( %) | 0% (262) |
| Neurological disorders, n (%) | 0% (262) |
| Ventilator-associated pneumonia, n (%)*** | 0% (155) |
| Duration of invasive mechanical ventilation (days)*** | 0% (155) |
| ICU length of stay (days) | 0% (262) |
|  |  |
| Outcomes |  |
| Outcomes in ICU, n (%) | 0% (262) |
| Outcomes at hospital discharge, n (%)** | 11% (78) |
| Outcomes at Day-30, n (%)** | 4% (84) |
| Outcomes at Day-90, n (%)** | 21% (69) |
|  |  |
| Variables are summarized as percentages of missing data (number of available data).  *Among the 16 patients with human immunodeficiency virus positive status.  **Among the 88 patients with lumbar puncture.  ***Among the 155 patients who were intubated.  Abbreviations: ICU: intensive care unit; SAPS: simplified acute physiology score; SOFA: sepsis-related organ failure assessment. | |
